# Pediatric glioma stem cells: biologic strategies for oncolytic HSV virotherapy

**DOI:** 10.3389/fonc.2013.00028

**Published:** 2013-02-28

**Authors:** Gregory K. Friedman, Joel Raborn, Virginia M. Kelly, Kevin A. Cassady, James M. Markert, G. Yancey Gillespie

**Affiliations:** ^1^Brain Tumor Research Program, Division of Pediatric Hematology and Oncology, Department of Pediatrics, University of Alabama at BirminghamBirmingham, AL, USA; ^2^School of Medicine, University of Alabama at BirminghamBirmingham, AL, USA; ^3^Division of Infectious Diseases, Department of Pediatrics, University of Alabama at BirminghamBirmingham, AL, USA; ^4^Division of Neurosurgery, Department of Surgery, University of Alabama at BirminghamBirmingham, AL, USA

**Keywords:** pediatric, glioblastoma, glioma stem cells, cancer stem cells, herpes simplex virus, HSV, oncolytic, virotherapy

## Abstract

While glioblastoma multiforme (GBM) is the most common adult malignant brain tumor, GBMs in childhood represent less than 10% of pediatric malignant brain tumors and are phenotypically and molecularly distinct from adult GBMs. Similar to adult patients, outcomes for children with high-grade gliomas (HGGs) remain poor. Furthermore, the significant morbidity and mortality yielded by pediatric GBM is compounded by neurotoxicity for the developing brain caused by current therapies. Poor outcomes have been attributed to a subpopulation of chemotherapy and radiotherapy resistant cells, termed “glioma stem cells” (GSCs), “glioma progenitor cells,” or “glioma-initiating cells,” which have the ability to initiate and maintain the tumor and to repopulate the recurring tumor after conventional therapy. Future innovative therapies for pediatric HGG must be able to eradicate these therapy-resistant GSCs. Oncolytic herpes simplex viruses (oHSV), genetically engineered to be safe for normal cells and to express diverse foreign anti-tumor therapeutic genes, have been demonstrated in preclinical studies to infect and kill GSCs and tumor cells equally while sparing normal brain cells. In this review, we discuss the unique aspects of pediatric GSCs, including markers to identify them, the microenvironment they reside in, signaling pathways that regulate them, mechanisms of cellular resistance, and approaches to target GSCs, with a focus on the promising therapeutic, genetically engineered oHSV.

## INTRODUCTION

Glioblastoma multiforme (GBM), classified as a high-grade malignant glioma, occurs in both adults and children. Although adult and childhood GBM are similarly heterogeneous tumors that develop from glial origin, significant differences exist between the tumors. While GBM is the most common malignant brain tumor in adults, it represents less than 10% of pediatric malignant brain tumors (Ries et al., 2006). Pediatric GBM typically arise *de novo* as a primary tumor whereas adult GBM may develop from the malignant progression of a low-grade glioma. Additionally, childhood high-grade gliomas (HGGs) can arise in the brainstem or spinal cord, which rarely occurs in adults. Despite multimodality therapy including surgery, chemotherapy, and radiotherapy, outcomes for both adults and children with HGGs remain poor with overall survival rates <20% (Massimino et al., 2005; Stupp et al., 2005; Song et al., 2010; Wolff et al., 2010; Cohen et al., 2011). Pediatric GBM patients have a marginal survival advantage compared to adults, however current therapies such as radiation can cause severe neurotoxicity to the developing brain that can further complicate the already significant morbidity in children. Many of the differences between pediatric and adult GBM may be attributed to distinct molecular patterns.

Integrated genomic analysis identified four clinically relevant subtypes of GBM in adults distinguished by gene aberrations such as *PDGFRα* and *IDH1* (proneural), *EGFR* (classical), and *NF1* (mesenchymal; Verhaak et al., 2010). The subtypes are not as well defined in pediatric GBM where genetic profiling revealed *PDGFRα* as the predominant focal amplification target and gene expression analyses indicated deregulation of *PDGFRα* signaling plays an important role in tumor development (Paugh et al., 2010). Furthermore, pediatric GBM demonstrate decreased expression of *EGFR* and reduced mutation rate of *IDH1* and *TP53* compared to adult GBM, whereas other molecular markers of poor prognosis such as MGMT overexpression and Akt activation remain similar (Pollack et al., 2001, 2006, 2010; Hegi et al., 2005; Paugh et al., 2010; MacDonald et al., 2011).

Glioblastoma multiformes are a heterogeneous mixture of numerous cell types, both neoplastic and non-neoplastic. Among the vascular, tumor, immune, and other various cell types, a subpopulation of critical cells exists termed “glioma stem cells” (GSCs; Singh et al., 2003; Galli et al., 2004). These GSCs are thought to have stem cell properties; they are multipotent and possess the ability to self-renew and to initiate and maintain the neoplastic clone. GSCs are putatively responsible for tumor initiation, maintenance, metastasis, and recurrence. Whether they are true stem cells has been debated with some preferring to call these cells “glioma progenitor cells” – suggesting the cells are more differentiated than a stem cell – or “glioma-initiating cells” which describes their ability to initiate tumors.

The origin of these cells and the triggers that result in their transformation are still being elucidated. GSCs, which share markers of normal neural stem cells, have been isolated from both pediatric low-grade gliomas (LGG) and HGGs suggesting that pediatric GSCs may emerge from normal neural stem cells that become mutated resulting in the loss of regulated cell division (Thirant et al., 2011). However, the origin of some GSCs may be a more committed cell; recent evidence suggests that even the most differentiated neurons and glial cells can dedifferentiate into stem-like cells and initiate gliomas (Friedmann-Morvinski et al., 2012). This indicates that there may be multiple cells of origin and this may result in clinical heterogeneity. Furthermore, because pediatric and adult gliomas are molecularly distinct, the initiating event resulting in a transformed GSC is likely different in children and adults. Irrespective of their origin, these cells have been implicated in the development of chemotherapy and radiation resistance which makes them clinically significant (Bao et al., 2006a; Eramo et al., 2006; Liu et al., 2006). Consequently, new innovative therapies are needed to target GSCs.

One such promising therapy is engineered oncolytic herpes simplex virus (oHSV) which has been genetically engineered to provide safety for normal cells but can target and kill cancer cells. In addition, a diverse range of foreign anti-tumor therapeutic genes can be expressed which augment the oncolytic effect and may specifically target unique features of GSCs. In this review, we discuss the unique aspects of pediatric GSCs, including markers to identify them, the microenvironment they reside in, signaling pathways that regulate them, mechanisms of cellular resistance, and approaches to target GSCs, with a focus on the promising therapeutic, genetically engineered oHSV.

## PEDIATRIC GLIOMA STEM CELLS

### MARKERS

Identifying GSCs is vitally important to study their behavior and function and to develop targeted therapies against them. GSCs, which share many of the characteristics of normal neural stem cells, have been identified by cell surface proteins, cytoplasmic and nuclear proteins, transcription factors, enzymes, and functional attributes (see **Table 1**; Dimov et al., 2011; Friedman and Gillespie, 2011). Hemmati et al. (2003) first recognized that when pediatric HGG cells were grown in culture in serum-free medium with specific stem cell defined growth factors including epidermal growth factor and basic fibroblast growth factor, the cells formed in clumps as spheres or “neurospheres” and expressed proteins normally associated with non-transformed neural stem cells such as CD133, the transcription factor Sox2, and the nuclear and cytoplasmic proteins musashi-1 and bmi-1. Subsequently, CD133 (prominin-1), a transmembrane protein with uncertain biologic function, became the most common marker used to identify cancer stem cells from a variety of diverse adult and pediatric solid tumors including HGGs.

**Table 1:** Markers used to identify glioma stem cells.

Attribute	Marker
Cell surface proteins	CD133, CD15, CD44, CXBR4, integrin alpha 6
Cytoplasmic and nuclear proteins	Nestin, musashi-1, bmi-1
Transcription factors	Sox2
Enzymes	ALDH1
Functional	Neurosphere formation, side population Tumor-initiating ability

While there are an abundance of studies utilizing CD133 to study GSCs in adult HGG models, few pediatric-specific studies exist. Shu et al. (2008) demonstrated that xenografts derived by implanting pediatric HGG cells orthotopically in immune-deficient mice could maintain the CD133^+^ GSC fraction, thus providing a useful model for studying GSCs. The CD133^+^ cells formed neurospheres and displayed multi-lineage differentiation capabilities in culture. In a pediatric GBM model, Donovan et al. (2012) found CD133^+^ cells expressed a higher percentage of the intermediate filament nestin, musashi-1, and CD15 (stage-specific embryonic antigen 1 or SSEA-1) and had increased proliferation compared to CD133^-^ cells. CD133 expression was directly linked to oxygen tension with increased expression seen in hypoxia, which is similar to our finding of a fourfold increase in CD133 expression in pediatric GBM xenograft cells maintained in stem cell defined medium and 1% hypoxia compared to normoxia (Friedman et al., 2012a). They found no difference in genomic imbalances in the CD133^+^ and CD133^-^ cells which is consistent with the transilience of CD133 expression observed. In an extensive examination of pediatric brain tumor surgical specimens, Thirant et al. (2011) found that tumor cells with stem-like properties could be isolated from both pediatric LGGs and HGGs. The cells from one LGG and several HGGs grew as neurospheres and shared a molecular profile of mesenchymal and neural stem cells including expression of Sox2, bmi-1, and nestin, suggesting a high level of plasticity. A majority of the cells in both the LGG and HGGs expressed CD133 and CD15.

Other cell surface markers which have been employed as an alternative to CD133 to identify GSCs in adult GBMs include CD15, which has also been established as a pediatric medulloblastoma stem cell marker; CD44, a cell adhesion and migration molecule; the adhesion molecule CXC chemokine receptor 4 (CXBR4); and intergrin alpha 6, the receptor for the extracellular matrix protein laminin (Mao et al., 2009; Read et al., 2009; Anido et al., 2010; Lathia et al., 2010; Zheng et al., 2011). Further studies are necessary to determine if these surface proteins are useful GSC markers in pediatric HGG. A number of other non-surface markers have been utilized to identify GSCs typically together with a surface marker. The cytoplasmic proteins musashi-1, an RNA-binding protein, and bmi-1, a polycomb protein, are thought to play a role in inhibiting differentiation and promoting self-renewal of GSCs (Okano et al., 2002; Abdouh et al., 2009). Sox2 maintains proliferation and inhibits differentiation of neural stem cells and possibly GSCs (Phi et al., 2008). Nestin, an intermediate filament found in neural stem cells and GSCs, appears to be involved in the structural organization of cells and important in cell proliferation and preventing differentiation (Michalczyk and Ziman, 2005; Dimov et al., 2011). Recently, the enzyme aldehyde dehydrogenase 1 (ALDH1) has been proposed as a GSC marker due to increased neurosphere formation and maintenance of tumor cells in an undifferentiated state with high levels of the protein (Rasper et al., 2010). Many studies have relied on functional attributes of cells such as the ability of cells to grow in neurospheres or to extrude Hoechst 33342 dye to identify a side population (SP) of cells (Wan et al., 2010). SP cells have increased expression of members of the ATP-binding cassette (ABC) transporters which play an important role in chemoresistance through rapid efflux of chemotherapeutics.

Many problems exist with the current methods to identify GSCs (Wan et al., 2010; Friedman and Gillespie, 2011). Defining and isolating populations of cells based on surface, cytoplasmic or nuclear markers, transcription factors, or enzymes can be challenging due to the transilient expression of some markers and the fact that expression is on a continuum and does not appear to be qualitatively associated with tumor cells. Thus, determining what is a positive GSC or negative tumor cell is often subjective and may explain discrepancies seen in the literature, such as reports of CD133^-^ cells representing the glioma-initiating cell population (Ogden et al., 2008; Wang et al., 2008; Chen et al., 2010; Prestegarden et al., 2010). Defining GSCs by cellular functional attributes is problematic as well for several reasons. The neurosphere assay is an *in vitro* cell culture phenomenon without a clear *in vivo* equivalence and without a standardized protocol. Single cells grown in serum-free medium will re-aggregate into neurospheres, and a majority of cells in a sphere may actually be differentiated tumor cells, making it unclear what population of cells is actually being studied. While SP identifies cells that may have inherent chemoresistance thereby making these cells clinically relevant, not all tumors contain a SP fraction and SP cells do not necessarily have stem-like properties. Therefore, new standardized methods to define, identify, isolate, and study GSCs are needed, and due to biologic differences in adult versus pediatric HGGs, studies conducted with pediatric models are required.

### NICHE

While defining GSC behavior and function is critical to designing therapies that can target these resistant cells, equally important is understanding the specialized microenvironment or niche where GSC reside and are maintained and regulated. Cell-to-cell communication, the extracellular matrix and tissue structure, various secreted factors and signals, and oxygen tension all influence the function of this poorly understood niche. GSCs are thought to reside in both hypoxic and perivascular niches which provide signals to GSCs that promote their maintenance and influence their behavior (Filatova et al., 2012). Similarly, GSCs produce factors that shape the niches. It is this cellular communication that appears to fosters tumor evolution and propagation; the crosstalk between cells has been shown to alter the microenvironment, such as stimulation of angiogenesis, and shape the recruitment of various cells from endothelial cells to immune cells, which can modulate antitumor responses, to normal neural stem cells which may become transformed (Filatova et al., 2012). GSCs appear to be regulated through a tight balance between inhibiting and promoting factors driven by various growth factors, cell-to-cell signaling and the extracellular matrix present in the perivascular compartment which maintain homeostasis or shift GSCs toward proliferation and differentiation (Bao et al., 2006b; Li and Neaves, 2006; Calabrese et al., 2007; Lathia et al., 2010).

A critical component of the niche and known regulator of GSCs is hypoxia. Gliomas experience a physiologic hypoxia with hypoxic gradients ranging from mild (10%) to moderate (2.5%) or severe (0.1%), which marks areas of necrosis, and HGGs contain more regions of moderate and severe hypoxia compared to LGGs (Collingridge et al., 1999; Evans et al., 2004a,b). Hypoxia supports GBM development, invasion and aggressiveness, angiogenesis, and resistance to chemotherapy and radiation (Evans et al., 2004a; Jensen, 2009), all features which may be due to hypoxia’s role in promoting the GSC-phenotype. Hypoxia has been shown to increase the pediatric GSC subpopulation and induce pediatric GSCs to secrete markedly elevated levels of vascular endothelial growth factor (VEGF; Bao et al., 2006b; Donovan et al., 2012; Friedman et al., 2012a). With the secretion of VEGF, GSCs promote endothelial cell migration and angiogenesis. Further elucidation of the complex role of the GSC niche in pediatric HGG is essential, so that future therapeutics can be designed to disrupt the microenvironment and thereby target GSCs.

### SIGNALING PATHWAYS

An important component of GSCs and their relationship with the microenvironment is the balance of signaling pathways which can further induce their stem-like properties. Normal signaling pathways and control mechanisms can become dysregulated through gene amplifications resulting in overexpression of receptors or ligands, or altered receptors that can initiate downstream pathways (Prenzel et al., 2001). The embryonic signaling pathways sonic Hedgehog (SHH), Wnt, and Notch have been shown to regulate normal neural stem cells and neurogenesis (Shi et al., 2008). Although currently there are few reports implicating these pathways in pediatric gliomas, studies have shown that these pathways actively promote the brain tumor stem cell-phenotype in pediatric medulloblastoma and ependymoma and in adult gliomas (Reya and Clevers, 2005; Fan et al., 2006; Shih and Holland, 2006; Clement et al., 2007; Yang et al., 2008; Moore, 2009; Nager et al., 2012). SHH has a critical role in the maintenance of GSCs by regulating stemness genes and promoting tumorigenesis and proliferation, and Hedgehog signaling is active in some pediatric diffuse intrinsic pontine gliomas (DIPGs; Clement et al., 2007; Monje et al., 2011; Takezaki et al., 2011). Inhibiting Hedgehog signaling prevented tumorigenicity and GSC proliferation in HGGs and decreased the self-renewal ability of DIPG neurospheres. The Wnt/β-catenin pathway has been linked to GSCs and their role in gliomagenesis and tumor proliferation and invasion (Nager et al., 2012). Notch signaling has been shown to enhance nestin expression, promote GSC self-renewal and radioresistance, and suppress GSC differentiation (Shih and Holland, 2006; Wang et al., 2010; Hu et al., 2011).

Receptor tyrosine kinases (RTKs) and other downstream pathways similarly have been causally linked to gliomagenesis and GSC maintenance and proliferation. The RTK family mediates several oncogenic growth factor pathways like EGFR and PDGFR that have been linked to malignancy, angiogenesis, self-renewal, and multipotency (Dimov et al., 2011). When GSCs were first discovered, Hemmati et al. (2003) established that epidermal growth factor was a critical components in medium used to culture and maintain GSCs. A recent study revealed that constitutively activated EGFRvIII expression and PTEN loss in murine neural stem cells resulted in glial tumors (Li et al., 2009). The transformed cells had increased proliferation, self-renewal ability, and CD133 expression. Jin et al. (2011) showed that EGFR signaling regulates GSC proliferation by inducing inhibitor of differentiation 3 (ID3)-driven cytokines. EGFR inhibitors decreased GSC neurosphere formation. While EGFR is more commonly amplified and overexpressed in adult gliomas, recent evidence suggests that EGFR amplification and EGFRvIII mutations may occur more often in pediatric HGGs than previously recognized (Bax et al., 2009). Downstream pathways of EGFR including phosphatidylinositol 3-kinase (PI3-K), Akt, and mTor likewise are critical regulators of GSCs. Inhibiting both PI3-K and mTor elicited a prodifferentiation effect on GSCs (Sunayama et al., 2010). Akt activation is common in pediatric HGGs and may represent a worse prognostic feature (Pollack et al., 2010). This may be due to Akt induced activation of transporter ABCG2 (Bleau et al., 2009).

PDGF overexpression has been implicated in gliomagenesis, and PDGFs can inhibit glial cell differentiation (Fomchenko and Holland, 2007). The effect of PDGF is not limited to autocrine function but also paracrine; production of PDGF by glioma cells can recruit glial progenitor cells and other cell types which play a key role in vasculogenesis and the tumor microenvironment (Assanah et al., 2006; Fomchenko and Holland, 2007). PDGFRα is overexpressed in most pediatric HGGs and gene amplification is seen in approximately one-third of HGGs including DIPGs (Paugh et al., 2010; Zarghooni et al., 2010). Further research into the role of various signaling pathways in pediatric GSCs and the GSC niche is needed, as these pathways represent promising treatment targets.

### RESISTANCE

A critical feature of GSCs which makes them clinically relevant, irrespective of their origin, is their inherent resistance to traditional chemotherapy and radiation. Several studies have demonstrated resistance of pediatric GSCs to chemotherapeutic agents like temozolomide and etoposide and to ionizing radiation (Bao et al., 2006a; Hussein et al., 2011; Thirant et al., 2011). The mechanisms proposed for the chemoresistance of pediatric GSCs include efficient DNA repair and high levels of expression of O^6^-methylguainine-DNA-methyltransferase (MGMT), cellular quiescence, and overexpression of multi-drug resistance transporters. Hussein et al. (2011) demonstrated that pediatric GSC neurospheres were able to repair DNA faster after exposure to the topoisomerase II inhibitor etoposide than glioma cells grown as a monolayer. Furthermore, cells grown as neurospheres were more likely to be in G_0_/G_1_ cell cycle phase than S or G_2_ suggesting that cellular quiescence may play a role.

MGMT, which removes alkyl groups from the O^6^ position of guanine thereby repairing DNA damage caused by alkylating agents like temozolomide, has been shown to be an important prognostic variable in pediatric GBM (Donson et al., 2007). Similar to adults, children with a methylated MGMT promoter which silences the gene and decreases the enzyme activity have a survival advantage compared to patients with an unmethylated promoter. Although no pediatric specific studies have examined MGMT status in GSCs, several adult studies have demonstrated that GSCs have high levels of MGMT and more efficiently repair DNA than non-GSC tumor cells (Liu et al., 2006; Pistollato et al., 2010). While MGMT is an important factor for temozolomide resistance in pediatric GBM, it is not an exclusive mechanism as an MGMT-independent mechanism has been identified. In a pediatric GBM cell line which was resistant to temozolomide in the absence of MGMT, Gaspar et al. (2010) discovered a PI3-K-mediated HOX/stem cell resistance gene signature. The resistant cell line was highly enriched for CD133 and revealed an up-regulation of *HOX* gene expression. Resistance to temozolomide was reversed with PI3-K pathway inhibition. Importantly, they showed a correlation between *HOXA9/HOXA10* gene expression in pediatric HGG samples and shorter patient survival. In embryonic development, *HOX* genes are critical for axis determination, and a differential expression of *HOX* genes has been found in neoplastic versus non-neoplastic human astrocytes (Abdel-Fattah et al., 2006). The exact mechanism by which *HOX* genes promote resistance to temozolomide is unclear, however it may be through an anti-apoptotic, pro-proliferative mechanism (Costa et al., 2010).

Pediatric GSCs are able to resist radiation damage by preferential activation of the DNA damage response (Bao et al., 2006a). Bao et al. (2006a) showed that the CD133^+^ fraction in D456MG, a pediatric GBM, was enriched after radiation, and the CD133^+^ GSCs survived radiation by more effectively repairing radiation-induced DNA damage compared to CD133^-^ cells. The radioresistance of the GSCs was reversed by inhibiting Chk1 and Chk2 checkpoint kinases. The adhesion molecule L1CAM (CD171) augmented the DNA damage checkpoint activation which increased radioresistance of GSCs by enhancing Chk2 signaling (Cheng et al., 2011). Due to the resistance of GSCs to conventional therapeutics and their role in tumor initiation, maintenance, metastasis, and recurrence, novel targeted agents that effectively kill GSCs are needed.

### TARGETING GLIOMA STEM CELLS

Strategies to target GSCs include direct attacks such as targeting unique biomarkers, blocking specific functions of GSCs, reversing or overcoming resistance mechanisms, or inducing differentiation; or indirect attacks at the GSC microenvironment (Binello and Germano, 2011; Friedman and Gillespie, 2011). The usefulness of an antibody targeted to a GSC marker depends on the specificity of that marker. While there are a wide variety of surface, cytoplasmic, and nuclear markers attributed to GSCs (**Table 1**), these markers are also found in normal cells. CD133, the most commonly used GSC maker, is expressed in neural, hematopoietic, and endothelial cells. Moreover, CD133 may not mark all GSCs and its expression is transilient making it a potentially less useful target. Newer GSC-specific biomarkers are needed which can be used for directed immune therapies and vaccinations, monoclonal antibodies, or small molecule inhibitors. Targeting specific functions of GSCs such as growth factors, developmental and signaling pathways and immune pathways is another promising approach (Binello and Germano, 2011). Various inhibitors have been or are being developed and tested, although there are a lack of studies in pediatric GBM models. Successful targeting of these pathways requires a comprehensive understanding of the regulators of each pathway, the interactions between pathways, and the role of these pathways in normal neural stem cell development.

As mechanisms by which pediatric GSCs resist traditional therapies are uncovered, they can be targeted to overcome that resistance or newer agents can be designed to militate against resistance mechanisms. Approaches include blocking DNA repair mechanisms such as disrupting the checkpoint response or targeting MGMT; or down-regulating various pathways, multi-drug resistance genes, or drug transporters involved in resistance. An alternative method is to induce differentiation of GSCs to tumor cells that are less tumorigenic and more sensitive to standard therapies. Isotretinoin is an example of this approach which has been used successfully in pediatric neuroblastoma patients and is currently undergoing a trial in children with medulloblastoma. In preclinical studies, retinoic acid differentiated GSCs, however obtaining terminal differentiation was not uniformly seen (Karsy et al., 2010; Niu et al., 2010).

Directly attacking GSCs may be limited by similarities of GSCs to normal neural stem cells as well as by the genomic instability of GSCs that may change their identity as a tumor progresses, therefore an alternative approach is indirectly targeting the GSC niche (LaBarge, 2010). This, too, poses many obstacles. The GSC niche must be distinct from the normal neural stem cell microenvironment to avoid damaging critical cells for normal tissue repair and maintenance. Furthermore, the niche may be unique in different areas of a tumor, in distant tumor sites, or in different stages of tumor progression requiring multiple approaches. In order to effectively target the GSC niche, an improved understanding of the residents and regulators of the niche is critical. Current methods to indirectly attack the GSC microenvironment include agents that target angiogenesis, the physiologic hypoxia experienced by HGG cells, or immune mediators of the niche (Binello and Germano, 2011). Few of these approaches have been tested in pediatric HGG models. Bao et al. (2006b) demonstrated that the anti-VEGF drug bevacizumab eliminated the proangiogenic effects of pediatric GSCs on endothelial cells and suppressed growth of GSC-derived xenografts. Bevacizumab is currently being studied in combination with temozolomide as adjuvant to surgical resection and radiation in children with GBM through the Children’s Oncology Group study ACNS0822. Alternatively, normal neural, mesenchymal, or embryonic stem cells that are attracted to the GSC microenvironment may be used as vehicles for delivery of various therapies such as cytokines, enzymes, or enzyme/prodrug suicide combinations to disrupt the niche and kill GSCs (Binello and Germano, 2012). No specific studies have examined these vehicles in pediatric GSCs, however human adipose mesenchymal stem cells that produce tumor necrosis factor-related apoptosis-inducing ligand (TRAIL) prolonged survival in animals with brainstem gliomas suggesting this may be a useful approach to treat DIPGs (Choi et al., 2011).

Another innovative promising approach to eradicate resistant pediatric GSCs is oncolytic virotherapy, which can be utilized to attack GSCs both directly and indirectly while sparing normal cells thereby limiting toxicities (Cripe et al., 2009; Friedman et al., 2012b). The direct mechanism of killing involves a virus infecting, replicating within, and lysing a GSC and then spreading to nearby cells. Candidate viruses either normally do not cause human disease but replicate in GSCs with altered signaling pathways or deficient interferon responses, or they contain mutations that prevent the virus from infecting or replicating in normal cells but permit infection and replication in GSCs. Large DNA viruses which contain many non-essential genes may be engineered to express therapeutic foreign gene products that can either directly target GSCs or indirectly target the GSC microenvironment. Genetically engineered oncolytic herpes simplex virus-1 (oHSV) is one such candidate virus that has effectively targeted GSCs directly and indirectly.

## ENGINEERED ONCOLYTIC HERPES SIMPLEX VIRUS

### ADVANTAGES AS A VIROTHERAPEUTIC AGENT

Oncolytic HSV offers several advantages as a virotherapeutic agent to target GSCs and pediatric HGGs. Herpes simplex virus-1 (HSV-1) is an enveloped, double-stranded (ds) DNA virus that does not integrate into the host cell DNA. The virus undergoes a cytolytic cycle which confers its oncolytic properties and this process is not cell cycle-dependent like traditional therapies. Thus, oHSV is able to evade many of the typical mechanisms that GSCs use to avoid killing by chemotherapy and radiation. This cycle initially involves cell entry via outer viral envelope glycoproteins B and C which facilitate the binding of glycoprotein D to one of several entry receptors including nectin-1 (CD111), nectin-2 (CD112), HVEM, and modified heparan sulfate proteoglycan (Spear, 2004). Once internalized, HSV moves to the cell nucleus where gene transcription occurs in a temporal fashion consisting of intermediate-early genes, followed by early genes and then late genes. The immediate-early proteins regulate expression of early genes, which encode enzymes necessary for viral DNA replication, and late genes, which encode proteins that form the structural proteins of the virion (Grandi et al., 2009). The envelope is derived by budding through the nuclear membrane of the cell, and the replication cycle ends with viral particles lysing the cell and spreading to nearby cells.

The HSV-1 genome is large (89 genes, 152 kb), and approximately 30 kb is “non-essential” for replication in cell culture. These genes while dispensable are important in viral pathogenesis *in vivo*. For the purposes of oHSV therapeutics, these non-essential genes can be replaced with foreign DNA for gene therapy to augment the oncolytic effect of the virus without compromising the virus’ ability to infect and replicate in glioma cells (Shah et al., 2003). Importantly, deletion of certain HSV genes (e.g., the γ_1_34.5 neurovirulence gene) facilitates selective replication in glioma cells (Chou et al., 1990; Markert et al., 1993). In the unlikely event that a mutant virus produces toxicity in normal brain tissue, effective antiviral agents are clinically available. Glial tumors are ideal targets for oHSV because the virus is neurotropic.

### CLINICAL USE AND SAFETY

Two viruses, G207 and HSV1716, have been safely injected intracerebrally in several phase I trials in adults with recurrent HGGs (see **Table 2** for a summary of viruses discussed in the text; Markert et al., 2000, 2009; Rampling et al., 2000; Papanastassiou et al., 2002; Harrow et al., 2004). G207 was derived from wild-type isolate HSV-1(F) strain. It contains deletions in both copies of the γ_1_34.5 gene and a *LacZ* gene insertion (which encodes β-galactosidase) into the U_L_39 locus thereby disabling expression of ICP6, the heavy chain for viral ribonucleotide reductase (RR; Mineta et al., 1995). The γ_1_34.5 gene encodes the 263 amino acid infected cell protein 34.5 (ICP34.5) which has two critical functions: (1) it is essential for HSV-1 neurovirulence enabling it to infect and replicate in neural cells, and (2) it enables the virus to overcome a cell’s normal host defense mechanism against infection. Importantly, its deletion also removes the opposite strand which encodes a portion of the latency-activated transcripts (LAT) making the γ_1_34.5-deleted HSVs less capable of establishing and/or reactivating from latency (Chou et al., 1990; Whitley et al., 1993; He et al., 1997).

**Table 2:** Summary of oncolytic HSVs discussed in the text.

Virus	Deletions	Foreign gene/promoter insertion	Reference
34.5ENVE	Deletions in γ_1_34.5 gene and in-frame gene-disrupting insertion of GFP within ICP6 gene	Vasculostatin (Vstat120) under control of the HSV IE 4/5 promoter and ICP34.5 under control of a synthetic nestin promoter	Yoo et al. (2012)
C134	Deletions in both copies of γ_1_34.5 gene	IRS1 gene under control of an HCMV immediate early promoter	Shah et al. (2007) Cassady et al. (2009)
G47Δ-mAngio	Deletions of the γ_1_34.5 and α47 genes and a disabling lacZ insertion within ICP6 gene	Murine angiostatin	Zhang et al. (2012)
G207	Deletions in both copies of γ_1_34.5 gene and disabling lacZ insertion within ICP6 gene	None	Hunter et al. (1999), Markert et al. (2000, 2009)
HSV1716	Deletions in both copies of γ_1_34.5 gene	None	Rampling et al. (2000), Papanastassiou et al. (2002), Harrow et al. (2004)
M002	Deletions in both copies of γ_1_34.5 gene	Murine IL-12 under the transcriptional control of the murine early-growth response-1 promoter (Egr-1)	Parker et al. (2000), Markert et al. (2012)
M032	Deletions in both copies of γ_1_34.5 gene	Human IL-12 under the transcriptional control of the murine early-growth response-1 promoter (Egr-1)	Markert et al. (2012)
MG18L	disabling lacZ insertion within ICP6 gene and a deletion at U_S_3	None	Kanai et al. (2011)
OncoVex^GM-CSF^	Complete deletions of the genes encoding ICP34.5 andICP47	GM-CSF, CMV promoter	Senzer et al. (2009)
OV-Chase	Deletions in both copies of γ_1_34.5 gene and in-frame gene-disrupting insertion of GFP within ICP6 gene	Chondroitinase ABC under control of the HSV IE4/5 promoter	Dmitrieva et al. (2011)
RAMBO	Deletions in both copies of γ_1_34.5 gene and in-frame gene-disrupting insertion of GFP within ICP6 gene	Vasculostatin (Vstat120) under control of the HSV IE 4/5 promoter	Hardcastle et al. (2010)
rQNestin34.5	Deletions in γ_1_34.5 gene and in-frame gene-disrupting insertion of GFP within ICP6 gene	ICP34.5 under control of a synthetic nestin promoter	Kambara et al. (2005a), Yoo et al. (2012)
VAE	Deletions in both copies of γ_1_34.5 gene	Endostatin-angiostatin fusion gene insert	Zhu et al. (2011)
Δ68H-6	Disabling lacZ insertion within ICP6 gene and γ_1_34.5 Beclin 1 binding domain deletion	None	Kanai et al. (2012b)

**FIGURE 1:**
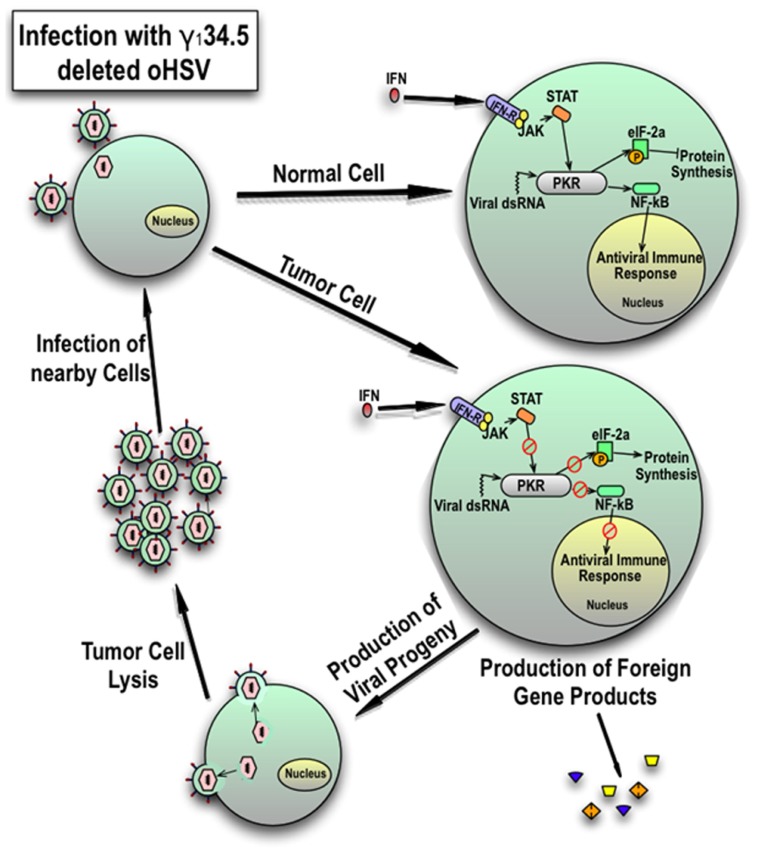
**Cellular response of normal cells and tumor cells to infection with γ_1_34.5-deleted HSV.** In response to viral dsRNA and interferon signaling, an intact PKR response in normal cells inhibits protein synthesis and consequently late viral gene expression by phosphorylation of eukaryotic initiating factor 2γ (eIF-2γ) and induces an antiviral response by activation of NFκB. However, in tumor cells, the PKR mediated response is blunted which results in late viral gene expression and reduced activation of the antiviral immune response. This results in production of viral progeny and expression of any added foreign genes. Resultant viral particles lyse the cell and spread to nearby tumor cells. Expression of foreign genes can be used to modulate the immune response or inhibit the microenvironment and tumor growth.

Herpes simplex virus infection leads to the production of non-host molecules that trigger a host-mediated antiviral response. The best characterized of these is the dsRNA-activated protein kinase R (PKR) translational arrest. After cell entry, HSV produces dsRNA. Upon binding viral dsRNA transcripts, PKR autophosphorylates and selectively phosphorylates eukaryotic initiating factor 2α (eIF-2α) which is essential for host protein translation in the infected cell (**Figure 1**; Williams, 2001). In wild-type HSV infection, the γ_1_34.5 product ICP34.5 binds and redirects protein phosphatase-1α which desphosphorylates eIF-2α and allows viral protein synthesis (He et al., 1997). Oncolytic HSVs deleted for the γ_1_34.5 gene are unable to evade PKR-mediated translational arrest thus limiting viral replication in normal cells containing a functional PKR response (Shah et al., 2007). Activated PKR also induces activation of nuclear factor-κB (NF-κB) which may contribute to antiviral responses (Taddeo et al., 2003). Mutant viruses with γ_1_34.5-deletions may replicate in tumor cells which have defective signaling pathways (e.g., defective PTEN and activated MAPK, both of which can be present in HGG), or activating *ras* mutations that results in defective PKR (Farassati et al., 2001; Smith et al., 2006). Both EGFR and PDGFR signaling, which are constitutively active in some pediatric HGGs, are known activators of *ras* (Schlessinger, 2000; Paugh et al., 2010; Zarghooni et al., 2010; Steelman et al., 2011).

The *lacZ* insertion adds additional protection for normal cells because RR is vital for nucleotide synthesis necessary for viral replication and mutants are hypersensitive to acyclovir (Goldstein and Weller, 1988; Coen et al., 1989). The insertion is less disruptive to virus replication in dividing cancer cells which can provide cellular RR for viral replication. Similar to G207, HSV1716 contains deletions in both copies of the γ_1_34.5 gene (also identified as R_L1_); however HSV1716 was derived from wild-type strain 17 which is a temperature insensitive isolate unlike the parental HSV-1(F) strain for G207 (MacLean et al., 1991). HSV1716 virulence is greater than that of G207 based on murine studies and the maximum safe dose [10^5^ PFU (plaque-forming unit) for 1716 vs. 3 × 10^9^ PFU for G207] used in clinical trials (Chambers et al., 1995; McKie et al., 1998).

G207 safety has been confirmed in both murine and non-human primate studies using New World owl monkeys (*Aotus nancymaae*) as young as 1-year old (Hunter et al., 1999). Owl monkeys are exquisitely sensitive to wild-type HSV-1 similar to human infants, and in the primate studies, inoculation with 1 × 10^3^ PFU of wild-type HSV-1(F) caused rapid mortality. In contrast, intracerebral inoculation with up to 1 × 10^9^ PFU of G207 (equivalent to a human dose of 5 × 10^10^ PFU) resulted in no short- or long-term clinical complications. Similarly, direct right frontal lobe inoculation in *A. nancymaae* of a γ_1_34.5-deleted HSV-1 M002, which has a foreign gene insert that expresses murine interleukin-12 (IL-12) in physiologic amounts, resulted in no clinical or magnetic resonance imaging (MRI) toxicity or histopathologic abnormalities at 1 month and 5.5 years after injection (Markert et al., 2012). These studies strongly suggest that γ_1_34.5-deleted viruses would be safe in children.

In a study by Radbill et al. (2007), neonatal mice at post-natal day 4 were injected intracerebrally with 2 μl of saline or 1 × 10^5^ PFU of G207. No difference was seen between the two groups in long-term physical development, cognitive performance, or exploratory behaviors suggesting the virus had no deleterious effect on neurodevelopment or brain function. The only abnormality seen was histological and MRI evidence of unilateral ventriculomegaly ipsilateral to the injection site in some of the G207-treated mice and in one saline-injected mouse that was attributed most likely to free-hand injection resulting in intraventricular delivery of the virus. The authors concluded that an initial study in children should exclude patients with intraventricular tumors and children under 2.

In the first adult clinical trial, G207 was proven safe with direct stereotactic inoculation of up to 3 × 10^9^ PFU of virus in up to five loci within enhancing portions of recurrent HGGs (Markert et al., 2000). There were no serious adverse events attributable to the virus, and therefore a maximum tolerated dose was not reached. A follow-up phase Ib study in adults with recurrent HGGs, likewise demonstrated safety of two separate stereotactic injections of G207 (total dose 1.15 × 10^9^; Markert et al., 2009). One dose was administered 2–5 days prior to resection and a second dose was subsequently given immediately after resection into the surrounding tumor cavity. While the studies were only designed to determine safety and not efficacy, responses were seen in a number of patients including two long-term survivors for >5.5 years. Patients with recurrent HGGs only live a few months on average. A third phase I trial of G207 injected into recurrent HGGs in five loci followed by a single 5 Gray fraction of ionizing radiation completed accrual and will be reported shortly. Low-dose radiation enhanced oHSV replication and efficacy in a preclinical HGG model suggesting the approach used in the trial may result in improved efficacy (Advani et al., 2011). This enhancement appears to be through multiple mechanisms including inhibition of radiation-induced DNA repair by HSV protein ICP0, up-regulation of cellular genes like RR that complement viral gene deletions, and greater activation of viral late promoters through the p38 mitogen-activated protein kinase pathway (Stanziale et al., 2002; Hadjipanayis and DeLuca, 2005; Mezhir et al., 2005).

Clinical trials of HSV1716 in HGG patients likewise demonstrated safety of the virus with inoculation intratumorally or into the tumor cavity (Rampling et al., 2000; Papanastassiou et al., 2002; Harrow et al., 2004). An antiviral immune response was documented in several patients, and no toxicity was seen in HSV-seronegative (or sero-positive) patients. Due to the more virulent nature of this HSV seen in mice, the highest dose examined was 1 × 10^5^ PFU. Importantly, like in the G207 trials, clinical responses were seen which resulted in several long-term survivors. While neither virus has been used in children with HGGs, HSV1716 is currently being used in a phase I trial in children older than 6 with recurrent solid tumors outside the central nervous system (ClinicalTrials.gov identifier: NCT00931931). The results of the G207 and HSV1716 trials indicate that further development of oHSV for the treatment of HGGs in both adults and children would be a worthwhile endeavor.

### oHSV TARGETING OF PEDIATRIC GSCs

For oHSV to have long-term efficacy in treating pediatric HGGs, the virus must be able to target and kill chemo- and radiation-resistant GSCs. GSCs have the ability to migrate through adjacent normal brain tissue, and this likely explains why many tumors recur directly outside the margins of radiotherapy. Thus, delivery and spread of oHSV is critical to target GSCs. Optimal delivery of oHSV to target dispersing GSCs remains to be defined. Intracerebral administration has been utilized to allow for direct inoculation of virus into the enhancing portions of a tumor or tumor bed by one or more passages of a stereotactically inserted needle or by passage of several neurosurgical catheters that have multiple openings near the tip followed by slow infusion by convection enhanced delivery over 6–12 h. The latter approach is more likely to provide a broader distribution of the virus. Theoretically, the infectious viral particles are small enough (150–200 nm) to percolate through the interstitial spaces in edematous brain, carried by convection of the transudate and CSF accumulating in the peritumoral spaces.

Another approach is systemic delivery of the virus; however this method is potentially complicated by the blood–brain barrier and HSV-1 seropositivity of the majority of adult humans which results in circulating antibodies that may deplete a significant amount of the intravenous dose. Since fewer children are sero-positive for HSV-1 (<20% positive in children under 4 and approximately 36% by age 13), children may be better candidate than adults for systemic therapy (Xu et al., 2007; Friedman et al., 2009b). The blood–brain barrier can be disrupted by osmotic solutions like mannitol or the inflammatory mediator bradykinin to permit intracarotid or intra-arterial injections of oHSV to target gliomas (Rainov et al., 1995; Liu et al., 2005). Other novel methods to delivery oHSV to brain tumors such as the use of carrier vehicles with inherent migratory and tumor tropic properties are currently being explored (Brown et al., 2003).

After an oHSV is delivered to a GSC, it must be able to enter the cell to kill it directly. While there are several entry receptors, nectin-1 (CD111) is the most efficient entry receptor for HSV-1 and has been shown to at least partially contribute to the variability in infectability of HGGs (Krummenacher et al., 2004; Rueger et al., 2005). We demonstrated that oHSV was unable to effectively enter and infect glioma xenograft cells with <20% nectin-1 expression (Friedman et al., 2009a). CD133^+^ GSCs expressed nectin-1 in similar amounts to CD133^-^ tumor cells in most GBM xenografts tested, including the pediatric GBM xenograft D456MG, which expressed high levels of nectin-1, suggesting that oHSV should be able to enter and infect GSCs and tumor cells equally. This was confirmed with cytotoxicity testing *in vitro*; the lethal dose required to kill 50% (LD_50_) of CD133^+^ or CD133^-^ D456MG cells was not significantly different, and D456MG, including the GSCs, were more sensitive to killing by G207 and M002 than six adult GBM xenografts tested (Cassady et al., 2009; Friedman and Gillespie, 2011). Athymic nude mice with D456MG implanted intracranially lived significantly longer after a single injection of the oHSV C134, a chimeric γ_1_34.5-deleted virus with the human cytomegalovirus (HCMV) IRS1 gene under control of the HCMV immediate early promoter; this insertion improves viral replication without restoring neurovirulence (Shah et al., 2007; Cassady et al., 2009). About 30% of the C134-treated mice were long-term survivors, suggesting that the GSC fraction was eradicated.

Interestingly, when GBM xenograft cells including D456MG were grown in 1% hypoxia to simulate the more severe physiologic hypoxic environment that glioma cells experience *in vivo*, nectin-1 expression increased significantly (12% increase in D456MG cells; Friedman et al., 2012a). Nectin-1 is an adhesion molecule and may be up-regulated in hypoxia to increase adhesion to vascular endothelial cells and facilitate tumor angiogenesis (Koike et al., 2004). Despite the increase in nectin-1, γ_1_34.5-deleted virus infectivity, replication, and cytotoxicity were significantly diminished in the pediatric GBM xenograft model under hypoxia. This suggests that other cellular factors can limit oHSV even if there are ample entry molecules.

**FIGURE 2:**
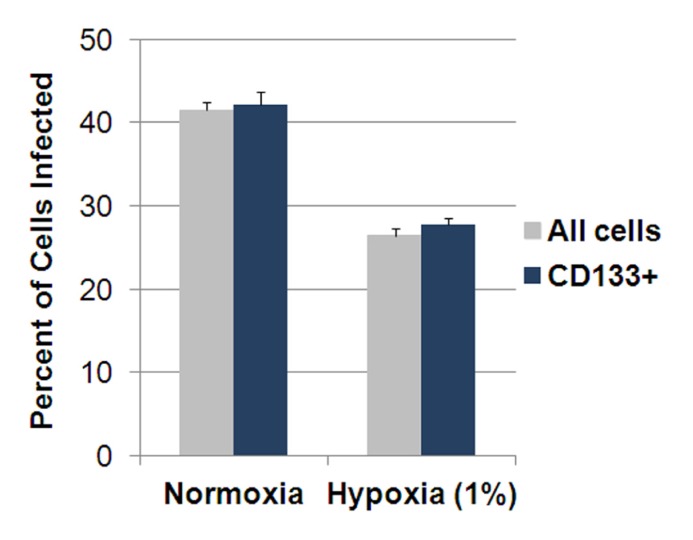
**Percentage of pediatric D456MG cells infected in normoxia and hypoxia by a γ_1_34.5-deleted virus 30 h after infection.** Cells were infected at 10 PFU/cell by a GFP-expressing γ_1_34.5-deleted virus for 30 h. After the addition of a CD133 allophycocyanin (APC) labeled antibody, cells were analyzed by fluorescent activated cell sorting (FACS) analysis looking for GFP and APC expression. There was a significant decrease in infectivity seen in all cells and CD133^+^ cells in hypoxia compared to normoxia but a similar number of CD133^+^ cells were infected compared to all cells in each environment suggesting CD133^+^ cells were not more resistant.

The decreased efficacy of a γ_1_34.5-deleted virus in physiologic hypoxic conditions is not inconsequential. As discussed earlier, hypoxia is a key regulator of GSCs by promoting the GSC-phenotype which supports GBM development, aggressiveness, angiogenesis, and resistance to chemotherapy and radiation (Jensen, 2009). While hypoxia significantly increased the CD133^+^ fraction in D456MG by nearly fourfold and decreased the percentage of CD133^+^ GSC infected, there was not a significant difference in the percentage of CD133^+^ cells infected compared to all tumor cells in normoxia or hypoxia suggesting that the CD133^+^ cells were not inherently more resistant (**Figure 2**; Friedman et al., 2012a). Nevertheless, a better understanding of the mechanism(s) behind the reduced γ_1_34.5-deleted virus efficacy in hypoxia is needed. Potential causes include reduced viral entry or transport, enhanced antiviral host cell responses, or increased autophagy limiting viral egress in hypoxia.

### FUTURE STRATEGIES FOR oHSV TARGETING OF PEDIATRIC GSCs

As GSC biology is being revealed, next-generation viruses are being developed and tested to directly and indirectly target GSCs through transcriptional or novel receptor targeting. Several approaches are being evaluated including retargeting the virus to infect GSCs; enhancing the efficacy of the virus by improving viral replication, harnessing the immune system, or attacking the GSC microenvironment; or combining oHSV with traditional therapies, small molecule inhibitors, or potentially other oncolytic viruses. As an example of transcriptional targeting, Kambara et al. (2005a) designed rQNestin34.5 which expresses ICP34.5 under control of a synthetic nestin promoter. As previously discussed, nestin is an intermediate filament protein expressed in GSC as well as embryonic and normal neural stem cells found in the subependymal zone in the brain, but it is not present in normal human astrocytes. While not tested specifically in GSCs, the virus prolonged survival in glioma-bearing mice. Whether or not this virus would also be able to replicate in normal neural stem cells is not clear.

Targeting other proteins that could serve as potential entry molecules that are specific for tumor cells but poorly expressed or not expressed in normal cells, Zhou and Roizman (2006) have devised a strategy of deleting the key recognition unit of glycoprotein D for CD111 and HVEM, but preserving the membrane fusion activity to allow virus entry. Inclusion of ligands for IL13-receptor α2 and urokinase plasminogen activator receptor has been shown to permit oHSV binding and entry into cells (Zhou et al., 2002; Zhou and Roizman, 2005; Kamiyama et al., 2006). Campadelli-Fiume and colleagues have exploited related strategies to insert a single chain antibody to bind Her2/neu (Lollini et al., 2009; Menotti et al., 2009; Gambini et al., 2012). Uchida et al. (2012) described another approach at retargeting oHSV by eliminating the natural receptor-binding activities of glycoprotein D; introducing single-chain antibodies specific for EGFR, which is overexpressed in many gliomas; and adding entry-accelerating mutations to glycoprotein B into a detargeted mutant form of glycoprotein D. The novel virus entered through EGFR and achieved infection similarly to wild-type virus while prolonging survival in mice with orthotopic primary human GBM xenografts. These studies highlight how oHSV may be used to specifically target GSCs.

While γ_1_34.5-deleted viruses can replicate in HGGs, replication is attenuated compared to wild-type HSV-1. Newer viruses have been designed which improve viral replication to achieve greater viral titers and to sustain infection longer in order to increase tumor cell transduction. The chimeric γ_1_34.5-deleted virus C134 restores one of the critical ICP34.5 functions of evading the host PKR antiviral response through production of HCMV IRS1 (Cassady, 2005). By restoring this function, late viral protein synthesis is augmented and higher viral titers are achieved in glioma cells without restoring neurovirulence. Compared to its parent γ_1_34.5-deleted virus, C134 was superior at improving survival in animals with gliomas (Shah et al., 2007). The virus is also very effective at killing D456MG GSCs (Cassady et al., 2009). C134 has been produced in clinical-grade for an upcoming trial in adults with recurrent HGGs, and a future pediatric trial is possible.

Autophagy, a catabolic process which involves the recycling of cellular contents and may lead to cellular self-degradation, plays an important role in the health of post-mitotic neuronal cells and in antiviral innate immunity by limiting viral production and thus spread to nearby cells (Orvedahl and Levine, 2008). Viral-induced PKR initiates autophagy through eIF-2α phosphorylation. Recent research suggests ICP34.5 confers neurovirulence by inhibiting autophagy through beclin-1 (Orvedahl et al., 2007). Kanai et al. (2012b) developed Δ68H-6, an ICP6 mutant with the γ_1_34.5 gene intact except for the Beclin-1 binding domain. The mutant virus was minimally neuropathogenic but replicated well in GSCs *in vitro* and prolonged survival in mice bearing orthotopic gliomas. These studies suggest that autophagy-related genes may be effectively manipulated to improve viral replication.

Another strategy which may improve the targeting of GSCs is to enhance the directed attack of the virus by employing the immune system. In addition to the attenuated replication of γ_1_34.5-deleted mutants, the tumoral environment of highly necrotic and infiltrative HGGs may limit the virus’ ability to infect all GSCs. One way to augment the antitumor effect of the virus is through cytokine production. In this model, the virus infects a GSC, replicates, and produces a cytokine of interest which attracts immune effector cells. The infected GSC is lysed by the virus which exposes GSC-specific antigens to the effector cells. This activates an antitumor immune response that leads to elimination of non-infected GSCs and tumor cells. Because effector cells may also eliminate the virus thus impeding its oncolytic effects, elucidating the right balance between virus replication and an antitumor response is likely critical (Alvarez-Breckenridge et al., 2012).

An example of a cytokine producing virus is M002. The virus produces physiologic relevant amounts of IL-12. IL-12 stimulates gamma interferon production from NK cells and T cells which activates cytotoxic T lymphocyte and T_h_1 cells. In addition, IL-12 promotes antiangiogenesis against tumor vasculature through NK cell activation and subsequent cytokine secretion (Parker et al., 2000; Hellums et al., 2005; Markert et al., 2012). In preclinical studies, M002 was safe and more efficacious than G207 and readily killed GSCs in a pediatric GBM model (Friedman et al., 2009a; Markert et al., 2012). The humanized IL-12 version of the virus, M032, has been produced in clinical-grade and a trial in adults with recurrent HGGs is forthcoming. Once safety in adults is confirmed, a pediatric trial will follow. A similar immune-enhanced 34.5-deleted virus, OncoVex^GM-CSF^, which produces clinically relevant amounts of granulocyte-macrophage colony-stimulating factor, showed efficacy in injected and uninjected lesions in some patients with unresectable metastatic melanoma and is currently being investigated in a phase III study (Senzer et al., 2009).

Indirectly targeting the GSC niche with oHSV gene therapy is another promising approach. Several unique viruses have recently been developed and tested preclinically, although none have been specifically tested in pediatric HGG models. The γ_1_34.5-deleted oHSV OV-Chase expresses chondroitinase ABC (Chase-ABC), a bacterial enzyme that removes the chondroitin sulfate from proteoglycans which are major constituents of the extracellular matrix of HGGs (Dmitrieva et al., 2011). Compared to its parent virus without Chase-ABC, OV-Chase significantly enhanced spread in glioma spheroids and prolonged survival in animals with intracranial glioma xenografts.

Several viruses which target angiogenesis have been evaluated. RAMBO (rapid antiangiogenesis-mediated by oncolytic virus) expresses vasculostatin (VStat120), the cleaved and secreted extracellular fragment of brain-specific angiogenesis inhibitor 1 (BAI1; Hardcastle et al., 2010). The virus inhibited glioma growth in mice and had an antiangiogenic effect as measured by a reduced tumor vascular volume fraction and microvessel density. A similar virus that expresses VStat120, 34.5ENVE, was created within the backbone of rQNestin34.5 and likewise prolonged survival in glioma-bearing mice and showed evidence of antiangiogenesis with reduced microvessel density and increased tumoral necrosis compared to a control virus (Yoo et al., 2012). Two angiostatin-based γ_1_34.5-deleted oHSVs have effectively targeted gliomas and GSCs. VAE carries an exogenous endo-angio fusion gene and produces the potent angiogenesis inhibitors endostatin and angiostatin (Zhu et al., 2011). Similar to the control virus, VAE-infected GSCs and significantly inhibited their viability and ability to self-renew. The fusion-gene was expressed in GSCs within 48 h after infection, and the semi-purified virally produced fusion protein reduced proliferation of brain microvascular endothelial cells *in vitro*, confirming the activity of the constituent proteins. Similarly, G47Δ-mAngio expresses angiostatin, and in combination with the VEGF inhibitor bevacizumab, the virus increased glioma tumor lysis and angiostatin-mediated inhibition of VEGF leading to a decreased invasive tumor phenotype (Zhang et al., 2012).

Similar to how combination chemotherapy strategies that use agents with different mechanisms of attack have enhanced tumor cell killing and decreased tumor resistance, combination approaches with oHSV and traditional therapies or small molecule inhibitors may improve outcomes and permit lower doses of toxic chemotherapeutics and radiation. This would limit toxicities, which is vital for potential use in the developing brain of a child. As previously discussed, low-dose radiation has been demonstrated to enhance oHSV replication (Advani et al., 2011). Recent studies have shown that temozolomide or low-dose etoposide and oHSV acted synergistically in killing GSCs (Cheema et al., 2011; Kanai et al., 2012a). The addition of cyclophosphamide to rQNEstin34.5 enabled lower doses of the virus to be used to achieve improved animal survival (Kambara et al., 2005b). Such combination strategies may allow for more virulent viruses to be used safely. New monoclonal antibodies and small molecule inhibitors may complement oHSV by augmenting viral replication, altering key regulatory pathways, and increasing apoptosis. MG18L, an oHSV with ICP6 disabled and deletion in U_s_3, which encodes a serine-threonine kinase with multiple functions including inhibition of virus-induced apoptosis and activation of Akt, acted synergistically with PI3-K/Akt inhibitors to target GSCs effectively (Kanai et al., 2011). There are an abundance of new antibodies and small molecule inhibitors that require testing in combination with oHSV.

### CONCLUDING REMARKS

Outcomes for children with HGGs remain poor with minimal improvement in overall survival rates seen over the past 30 years, and current therapies are extremely toxic to the developing brain. The difficulty in curing children with HGGs is likely due to a chemotherapy and radiation-resistant population of cells that play an integral role in tumor initiation, proliferation, invasiveness, and aggressiveness. These GSCs are maintained by a variety of signaling pathways and reside in a tightly regulated microenvironment. Novel therapeutics which can target and kill GSCs are desperately needed to improve outcomes and reduce morbidity in children with HGGs.

oHSV, which can directly attack GSCs and directly and indirectly target them through expression of various therapeutic foreign genes, offers an innovative, safe approach. A variety of strategies to enhance viral efficacy are being developed such as improving delivery methods, tumor specificity, and viral replication; minimizing viral clearance and antiviral defense mechanisms; increasing tumor-directed immune responses; and disrupting the tumor microenvironment. Combining these various strategies will likely result in more efficacious viruses, and combining oHSV with radiation, various chemotherapeutics including monoclonal antibodies and small molecule inhibitors, and possibly even other viruses with different mechanisms of attack will likely provide a synergistic effect. Through these various combination approaches, oHSV has great potential to target resistant GSCs thereby improving outcomes and quality of life by decreasing toxicity for children with HGGs.

## Conflict of Interest Statement

Dr. G. Yancey Gillespie and Dr. James M. Markert are stockholders, principal founders, and consultants for Catherex, Inc, a biotech company that holds extensive intellectual property in, and seeks to advance clinical application of oncolytic HSV therapy for cancer.
